# Diminished Systemic Amino Acids Metabolome and Lipid Peroxidation in Ureteropelvic Junction Obstruction (UPJO) Infants Requiring Surgery

**DOI:** 10.3390/jcm10071467

**Published:** 2021-04-02

**Authors:** Olga Begou, Antigoni Pavlaki, Olga Deda, Alexander Bollenbach, Kathrin Drabert, Helen Gika, Evangelia Farmaki, John Dotis, Nikoleta Printza, Georgios Theodoridis, Dimitrios Tsikas

**Affiliations:** 1 Laboratory of Analytical Chemistry, Department of Chemistry, Aristotle University of Thessaloniki, University Campus, 54124 Thessaloniki, Greece; gtheodor@chem.auth.gr; 2 Biomic_AUTh, Center for Interdisciplinary Research and Innovation (CIRI-AUTH), Balkan Center, B1.4, 10th km Thessaloniki-Thermi Rd, P.O. Box 8318, 57001 Thessaloniki, Greece; oliadmy@gmail.com (O.D.); gkikae@auth.gr (H.G.); 3 Core Unit Proteomics, Hannover Medical School, Institute of Toxicology, Carl-Neuberg-Strasse 1, 30625 Hannover, Germany; bollenbach.alex@gmail.com (A.B.); drabert.kathrin@mh-hannover.de (K.D.); tsikas.dimitros@mh-hannover.de (D.T.); 4 Paediatric Nephrology Unit, First Department of Paediatrics, Hippokratio Hospital, Aristotle University of Thessaloniki, University Campus, 54124 Thessaloniki, Greece; antigonipavlaki@yahoo.gr (A.P.); yan_dot@yahoo.com (J.D.); nprintza@auth.gr (N.P.); 5 Laboratory of Forensic Medicine and Toxicology, School of Medicine, Aristotle University of Thessaloniki, University Campus, 54124 Thessaloniki, Greece; 6 Paediatric Immunology and Rheumatology Referral Center, First Department of Paediatrics, Hippokratio Hospital, University Campus, 54124 Thessaloniki, Greece; farmakg@auth.gr

**Keywords:** GC-MS, amino acids, UPJO, serum, neonates, biomarkers, patient stratification

## Abstract

Congenital anomalies of the urinary tract, and particularly of obstructive nephropathy such as ureteropelvic junction obstruction (UPJO) in infants, can later lead to chronic kidney disease and hypertension. Fundamental questions regarding underlying mechanisms remain unanswered. The aim of the present study was to quantitate the systemic amino acids metabolome in 21 UPJO infants requiring surgery (Group A) and 21 UPJO infants under conservative treatment (Group B). Nineteen healthy age-matched infants served as controls (Group C). Serum amino acids involved in several pathways and representative metabolites, including the L-arginine-derived nitric oxide (NO) metabolites nitrite and nitrate and the lipid peroxidation biomarker malondialdehyde (MDA) were measured by gas chromatography–mass spectrometry (GC–MS) methods using their stable-isotope labeled analogs as internal standards after derivatization to their methyl esters *N*-pentafluoropropionic amides (amino acids) and to their pentafluorobenzyl derivatives (nitrite, nitrate, MDA). The concentrations of the majority of the biomarkers were found to be lower in Group A compared to Group B. Statistical analysis revealed clear differentiation between the examined study groups. Univariate statistical analysis highlighted serum homoarginine (*q* = 0.006), asymmetric dimethylarginine (*q* = 0.05) and malondialdehyde (*q* = 0.022) as potential biomarkers for UPJO infants requiring surgery. Group A also differed from Group B with respect to the diameter of the preoperative anterior–posterior renal pelvis (AP) as well as regarding the number and extent of inverse correlations between AP and the serum concentrations of the biomarkers. In Group A, but not in Group B, the AP diameter strongly correlated with hydroxy-proline (*r* = −0.746, *p* = 0.0002) and MDA (*r* = −0.754, *p* = 0.002). Our results indicate a diminished amino acids metabolome in the serum of UPJO infants requiring surgery comparing to a conservative group.

## 1. Introduction

One of the most common abnormalities detected upon prenatal ultrasound examination is hydronephrosis [1]. Although 50–70% of prenatally diagnosed hydronephrosis will be proven transient, the majority of the remaining cases will be attributed to obstructive uropathies, and ureteropelvic junction obstruction (UPJO) remains the commonest of those [2], with its frequency to be estimated at 1:1500 newborns [3,4].

The management of a patient who suffers from mild or severe hydronephrosis is considered “easier” since the clinical outcome is usually predictable. Mild hydronephrosis improves or stabilizes in 98% of the cases [5]. Children suffering from severe hydronephrosis will require surgical intervention in almost 100% of the cases [5]. On the other hand, patients suffering from moderate hydronephrosis present with a significant variability of clinical outcomes. Given the wide variation in the clinical course of children with moderate hydronephrosis, there is no unanimity on the criteria for surgical treatment [6]. Early detection of hydronephrosis has reduced cases of severe renal damage due to obstruction or infections. However, many of the prenatally diagnosed cases that will resolve spontaneously will be subjected to unnecessary and costly investigations. The challenge remains the following: How to predict at an early stage which of these prenatally detected infants will actually benefit from surgical treatment before irreversible renal damage is established.

Findings from already published studies regarding biomarkers and their utility in hydronephrosis management are often conflicting, and none of them has been incorporated in regular clinical practice to date [7]. Most experts conclude that further multi-disciplinary and multi-institutional studies are needed in order to identify a marker or a panel of markers that could reliably identify obstructed renal units that are at risk of deterioration [7,8].

So far, few metabolomics-based studies have been conducted evaluating UPJO in either human cohorts [9,10] or rodents [11,12,13,14,15,16,17,18]. Boizard et al. identified 32 features with differential urine abundance and managed to construct a prediction model that allowed them to diagnose UPJO cases with 76% sensitivity and 86% specificity [9]. Our group published a prospective case-control study in which the serum metabolic profile of surgical cases of infants with UPJO was compared to both nonsurgical cases and healthy age-matched controls. Hydrophilic interaction liquid chromatography (HILIC)-based liquid chromatography coupled with mass spectrometry (LC–MS/MS) was employed as the analytical tool. Principal component analysis (PCA) and orthogonal partial least squares-discriminant analysis (OPLS-DA) score plots showed that the studied groups differed significantly, with a panel of circulating metabolites including creatinine, tryptophan, choline and aspartate distinguishing patients who required surgery from those followed by systematical monitoring as well as from healthy controls [10].

Previous studies suggested that children with UPJO have increased expression and activity of inducible nitric oxide synthase (iNOS) and endothelial nitric oxide synthase (eNOS) in the medulla and cortex, respectively, presumably due to inflammatory processes which are commonly associated with elevated oxidative stress [19,20,21]. iNOS and eNOS catalyze the conversion of L-arginine to nitric oxide (NO), which is a major regulator of multiple organs, including the kidney. Yet, the precise role of the L-arginine/NO pathway in pediatric UPJO remains elusive. In the kidney, additional more abundant L-arginine-involving pathways such as those generating creatine and homoarginine play important roles in health and disease, but their importance in pediatric UPJO is also poorly investigated. In the present study, gas chromatographic–mass spectrometric (GC–MS) metabolomics-based methods were employed for the quantitative determination in the serums of newborn and infants with UPJO of amino acids and their metabolites as well as malondialdehyde (MDA), an established biomarker of lipid peroxidation [22]. The aim of the study was to identify the potential diagnostic and prognostic value of biomarkers of the above-mentioned arginine-involving pathways in pediatric UPJO.

## 2. Materials and Methods

### 2.1. Chemicals, Reagents and Materials

All amino acids were obtained from Sigma (Deisenhofen, Germany). 1,1,3,3-Tetraethoxypropane, 2,3,4,5,6-pentafluorobenzyl bromide (PFB-Br), pentafluoropropionic anhydride (PFPA, >99%) and the sodium salts (^15^N)nitrite) and (^15^N)nitrate (each 99 atom% ^15^N) were purchased from Sigma-Aldrich (Steinheim, Germany). Ethyl acetate (EA) and acetone were obtained from Baker (Deventer, The Netherlands), boric acid was purchased from Merck (Darmstadt, Germany) and toluene from Sigma (Deisenhofen, Germany), while methanol (MeOH, UHPLC grade) and hydrochloric acid (HCl, 37%) were obtained from Mallinckrodt Baker (Griesheim, Germany). Tetradeuterated methanol (CD_3_OD, 99 atom% ^2^H) was supplied by Aldrich (Steinheim, Germany). [1,3-^2^H_2_]-1,1,3,3-tetraethoxypropane (>95 atom% ^2^H) was obtained from Cambridge Isotope Laboratories (Andover, MA, USA) Endogenous unlabeled malondialdehyde (d_0_-MDA) and deuterium-labeled malondialdehyde (1,3-d_2_-malondialdehyde, d_2_-MDA) were prepared from HCl-catalyzed hydrolysis of commercially available 1,1,3,3-tetraethoxypropane and [1,3-^2^H_2_]-1,1,3,3-tetraethoxypropane, respectively, as described elsewhere [23]. d_2_-MDA served as the internal standard for d_0_-MDA. Anhydrous sodium sulfate was obtained from Merck (Darmstadt, Germany).

### 2.2. Study Population and Serum Collection

The demographic and clinical characteristics of the subjects involved in the study are summarized in Table 1. Patients participating in the present study constitute a subgroup of a cohort, members of whom have participated in three previously published studies [10,24,25].

Neonates and infants with unilateral hydronephrosis (antenatal or not) were screened for vesicoureteral reflux, and if found negative, they entered the protocol. Patients were excluded from the study if they had a positive urine culture, a history of bilateral UPJO, bilateral hydronephrosis, any other obstructive uropathy (such as vesicoureteral junction obstruction, posterior urethral valves, or malformations in the lower ureter), urinary stones, autoimmune disease, immunodeficiency, previous surgery of the urinary system, or any kind of malformation of the external genitalia.

Healthy, age-matched neonates and infants were recruited as controls supposing that their personal and family history was free from any kind of kidney disease and that they presented with a normal antenatal ultrasound scan.

For each participant in the study, epidemiological and clinical data were recorded. The results of renal ultrasounds and diuretic technetium-99m mercaptoacetyltriglycine (Tc-99m-MAG-3) renographies were also recorded. The degree of hydronephrosis was graded according to the Society of Fetal Urology (SFU) system [26]. In accordance with the declaration of Helsinki, after an explanation of the purpose of the study, written informed consent was obtained from all parents. The present research protocol was approved by the Bioethics Committee of Aristotle University of Thessaloniki (422/9.5.18).

Based on the results of the above tests and in accordance with international standard criteria [27], infants with obstructive UPJO were selected and referred for surgery. Consequently, the original group of participants was divided into two groups: Group A consisted of 21 UPJO patients (19/2, male/female) suffering from obstructive UPJO and being treated surgically; Group B included 21 patients (16/5, male/female) who presented nonsurgical UPJO and were treated conservatively. Group C recruited 19 healthy aged-matched newborns and infants (11/8, male/female) and served as a control group. No statistically significant differentiation was observed regarding age of diagnosis, as most infants presented with an antenatal diagnosis, while median age of admission in the study was 2 months for all groups. Renal pelvis anterior–posterior diameter (AP) appeared to be higher in Group A compared to Group B, as expected (*p* value 0.02). Serum creatinine (SCr) values were within normal ranges for all participants and did not differ significantly between the three groups (Table 1).

Serum samples were collected preoperatively from surgical UPJO patients (Group A), at initial diagnosis from UPJO patients placed on systematic monitoring (Group B), and from healthy controls (Group C). All serum samples were kept frozen at −80 °C until analysis.

### 2.3. Gas Chromatography–Mass Spectrometry Analyses

The concentrations of all analytes in study serum samples were measured by stable-isotope dilution gas chromatography–mass spectrometry (GC–MS) using previously developed and validated methods [28,29]. GC–MS was performed on a single quadrupole mass spectrometer model ISQ coupled with a Trace 1210 series gas chromatograph and an AS 1310 autosampler from ThermoFisher (Dreieich, Germany). A fused-silica capillary column Optima 17 (15 m, 0.25 mm I.D., 0.25 μm film thickness) from Macherey-Nagel (Düren, Germany) was used. Quantification was performed by selected-ion monitoring (SIM) the characteristics of mass–to–charge (*m*/*z*) ions of unlabeled and stable-isotope labeled analogs that served as internal standards (Table S1). To assess the precision of the GC–MS methods, quality control was performed [30,31]. The quality control (QC) sample was prepared by mixing equal volumes of all serum samples of the study. In total, 12 QC samples for amino acids and 7 QC samples for nitrate, nitrite and MDA were analyzed concomitantly with the study serum samples. The procedures for sample preparation, derivatization and GC–MS analyses and the results of the QC are reported in the Supplementary Materials.

### 2.4. Data Handling—Statistical Analyses

Raw chromatographic data obtained from the GC–MS analyses and peak integration of all detected endogenous analytes and their respective internal standards were performed in Xcalibur™ software 2.2 (Thermo Fisher Scientific™, Waltham, MA, USA). Quantification of all samples was conducted using Microsoft Excel (Microsoft Office, Redmond, WA, USA).

Statistical data analyses were performed using the advanced statistical software GraphPad Prism 7.0 for Windows (GraphPad Software, La Jolla, CA, USA). Data were checked for a normal distribution based on D’Agostino-Pearson (95% *de*). In addition, one-way ANOVA followed by post-hoc analysis and false discovery rate (FDR) correction (Benjamini, Krieger, and Yekutieli) were conducted. The threshold value was set at 0.05 (*q* value). Area under the curve–receiver operating characteristic (AUC–ROC) curves and box plots were extracted for the statistically significant metabolites. The logarithm base two of fold change (log2FC; FC, fold change) were calculated as the ratio of the final value (UPJO groups) over the initial value (control group). The correlation of biomarkers and other biochemical parameters was assessed using Pearson or Spearman correlation, depending on data distribution.

## 3. Results

The aim of the present study was to investigate potential differentiation between serum metabolic profiles of L-arginine-involving pathways and lipid peroxidation, possibly allowing identification of early diagnostic and prognostic markers of UPJO in infants. Serum samples of 42 neonates and infants with UPJO and 19 age-matched healthy controls were analyzed by GC–MS for the quantitative determination of several amino acids and their metabolites, especially including the L-arginine/NO pathway, which has been reported to play a role in UPJO [19,20,21]. Nitrite and nitrate were used as surrogates for NO, which is not detectable in blood [32]. In addition, we also measured MDA as an established biomarker of oxidative stress: Notably, lipid peroxidation of polyunsaturated fatty acids [22]. All parameters were measured by previously reported and validated GC–MS methods [28,29]. Exemplary chromatograms from GC–MS analyses of amino acids (Figure S1A), nitrite, nitrate and MDA (Figure S1B) in quality control serum samples are shown in Figure S1. The derivatives of leucine and isoleucine co-elute, while the derivatives of asparagine and aspartate, glutamine and glutamate, as well as ornithine and citrulline elute each as a peak due to conversion of asparagine to aspartate, glutamine to glutamate and citrulline to ornithine during the first derivatization step (esterification), respectively. For these amino acids, their sums were determined and reported.

### 3.1. Study Serum Concentrations of Amino Acids, Their Metabolites, and Malondialdehyde

**Figure 1:**
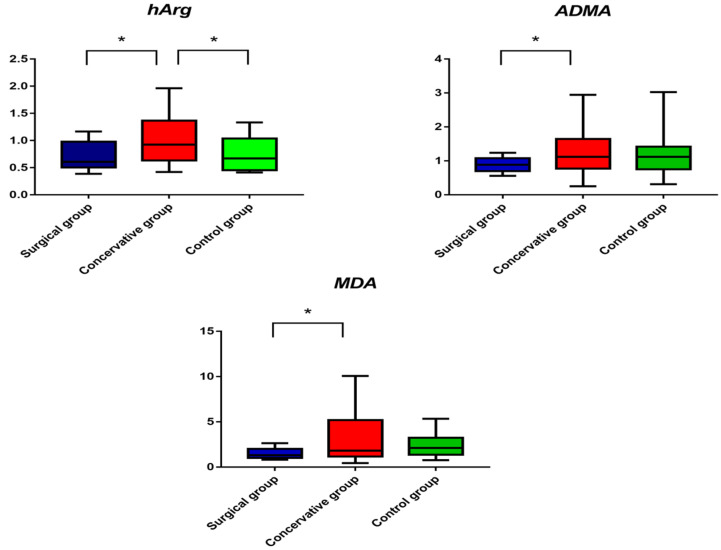
Box plots of serum concentrations of homoarginine (hArg), asymmetric dimethylarginine (ADMA) and malondialdehyde (MDA) in the three study groups. Data are plotted as median with range. An asterisk indicates statistical significance (*q* < 0.05).

**Figure 2:**
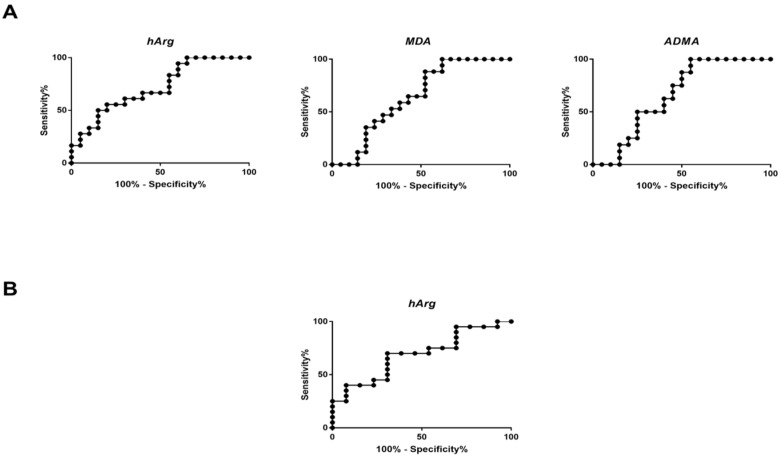
ROC curves of serum homoarginine (hArg), malondialdehyde (MDA) and asymmetric dimethylarginine (ADMA) and between comparisons of (**A**) group A vs. group B, and (**B**) between comparison of group B and group C for hArg.

Table 2 presents the median serum concentrations of all analytes in the three examined groups as measured by GC–MS. The data was subjected to further statistical analysis. One-way ANOVA suggested a statistically significant alteration in homoarginine (hArg) between groups A and B (*q* value = 0.006) as well as between groups B and C (*q* value = 0.014). Asymmetric dimethylarginine (ADMA) also presented significantly lower median serum concentration in the surgical group compared to the conservative one (*q* value = 0.05). In all cases, serum concentrations of hArg, ADMA and MDA were higher in Group B compared to group A. Figure 1 and Figure 2A illustrate box plots of hArg and ADMA and the AUC–ROC curves in the examined groups, respectively. Irrespective of statistical significance, a series of metabolites showed significant fold change above 20%. Specifically, the serum concentrations of alanine, threonine, serine, sarcosine, glutamine + glutamate, ornithine + citrulline and tyrosine were >20% higher in Group B compared to Group A. Also, the serum concentrations of threonine, sarcosine and ADMA were lower by 20% in Group A compared to Group C. Those of alanine, guanidino acetic acid and arginine were higher in Group B compared to the control Group C, while only threonine was lower by 20% in the same comparison.

Univariate statistical analysis of the obtained data and one-way ANOVA combined with post hoc analysis and FDR correction showed statistically significant differentiation in MDA between surgical and conservative groups (1.5 μM vs. 3.4 μM, *q* value = 0.022). Median serum concentrations of Group B were higher compared to Group A and Group B. Figure 1 illustrates the box plots of MDA in the three groups examined. Figure 2B presents the AUC–ROC curve of the comparison between Group A and Group B. Table 3 summarizes all significant metabolites along with their respective log2FC and AUC–ROC values.

### 3.2. Correlations between Serum Metabolites

**Figure 3:**
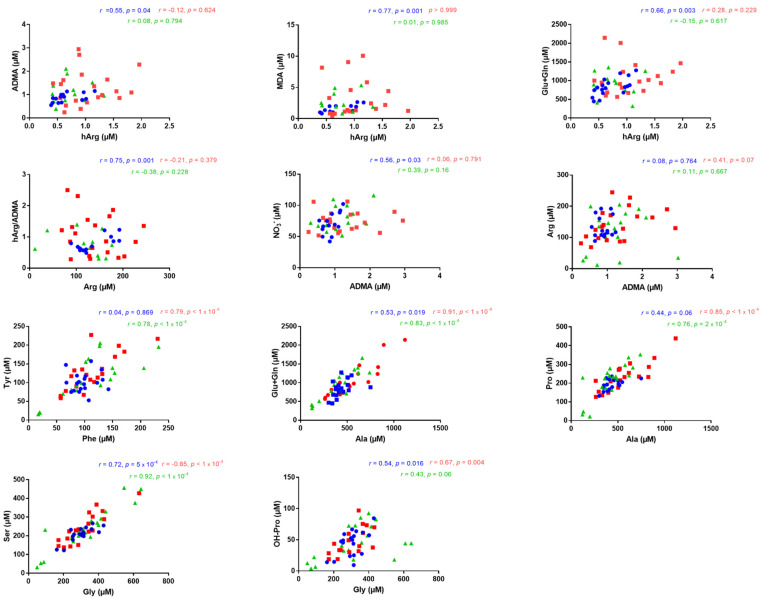
Illustration of statistically significant correlations (*r* > 0.4, *p* < 0.05) found between the serum concentrations of the indicated metabolites in Group A (surgical treatment) (blue), in Group B (conservative treatment) (red) and in Group C (control group) (green). Pearson or Spearman correlation was performed depending on data distribution.

Possible correlations between the serum concentrations of the analytes measured by GC–MS were tested in the study Group A (surgical treatment) and in the study Group B (conservative treatment). In Group A, positive correlations were found between hArg and ADMA (*r* = 0.55, *p* = 0.04), hArg and MDA (*r* = 0.77, *p* = 0.001) and hArg and Glu + Gln (*r* = 0.66, *p* = 0.003) (Figure 3). In Group A, the serum hArg/ADMA molar ratio correlated with the serum Arg concentration (*r* = 0.75, *p* = 0.001), and nitrate correlated with ADMA (*r* = 0.56, *p* = 0.03). Statistically significant correlations were also found between the analytes Ala and Glu + Gln (*r* = 0.53, *p* = 0.019), Gly and Ser (*r* = 0.72, *p* = 5 × 10^−4^) and Gly and OH-Pro (*r* = 0.54, *p* = 0.016) (Figure 3). No correlation was observed between the serum concentrations of nitrite and nitrate (data not shown). In Group B, a very borderline correlation was found between the serum concentrations of ADMA and Arg (*r* = 0.41, *p* = 0.07). In the same group, strong to very strong correlations were found between metabolically related amino acids: Phe and Tyr (*r* = 0.79, *p* < 1 × 10^−4^), Ala and Glu + Gln (*r* = 0.91, *p* < 1 × 10^−4^), Ala and Pro (*r* = 0.85, *p* < 1 × 10^−4^), Gly and Ser (*r* = 0.85, *p* < 1 × 10^−4^) and Gly and OH-Pro (*r* = 0.67, *p* = 0.004) (Figure 3). The same pairwise correlation of amino acids was also estimated in the control group (Figure 3). The differences between the groups regarding these correlations could in part be due to differences in amino acid management by the infants.

### 3.3. Correlations between Serum Metabolites and Clinical Data

**Figure 4:**
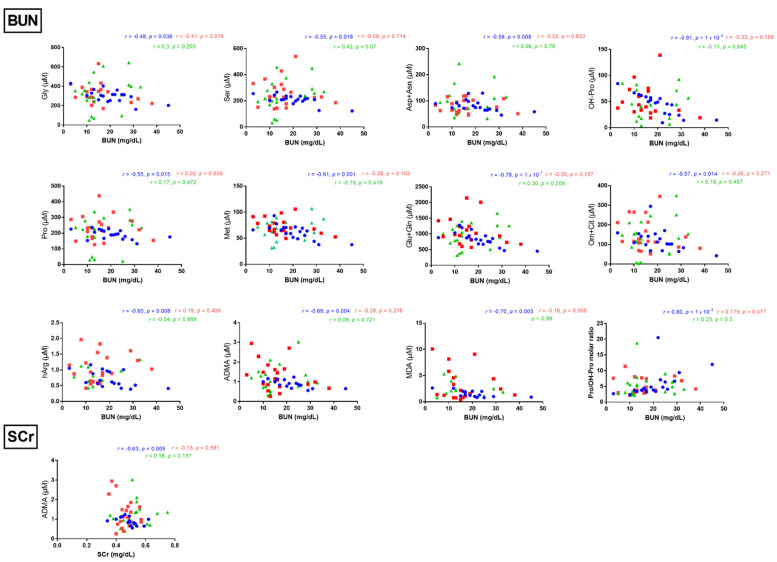
Illustration of statistically significant correlations (*r* > 0.4, *p* < 0.05) found between blood urea nitrogen (BUN) or serum creatinine (SCr) and the serum concentrations of indicated metabolites in Group A (surgical treatment). Spearman correlation was performed in all comparisons.

Several correlations were performed between serum metabolites and some other biochemical parameters such as blood urea nitrogen (BUN), serum creatinine and the patients’ anthropometric characteristics including gestational age and birth weight. In Group A, BUN was inversely correlated with several metabolites, namely Gly (*r* = −0.48, *p* = 0.036), Ser (*r* = −0.54, *p* = 0.018), Asp + Asn (*r* = −0.59, *p* = 0.008), OH-Pro (*r* = −0.91, *p* < 1 × 10^−4^), Pro (*r* = −0.55, *p* = 0.015), Met (*r* = −0.91, *p* = 0.0007), Glu + Gln (*r* = −0.78, *p* < 1 × 10^−4^), Orn + Cit (*r* = −0.57, *p* = 0.014), hArg (*r* = −0.60, *p* = 0.008), ADMA (*r* = −0.69, *p* = 0.004) and MDA (*r* = −0.70, *p* = 0.005), and correlated positively only with the serum Pro/OH-Pro molar ratio (*r* = 0.80, *p* < 1 × 10^−4^) (Figure 4). In addition, in Group A, creatinine was found to be inversely correlated with serum ADMA correlation (*r* = −0.63, *p* = 0.009) (Figure 4). However, in Group B, no correlation was observed between BUN and serum metabolites. Regarding patients’ anthropometric characteristics, birth weight did not correlate with the measured analytes, whereas gestational age correlated negatively only with nitrite in Group A (*r* = −0.71, *p* = 0.0006). In the control group, no significant correlations were observed between BUN or SCr and measured analytes (Figure 4).

### 3.4. Correlations between Anterior–Posterior Diameter and Serum Metabolites

**Figure 5:**
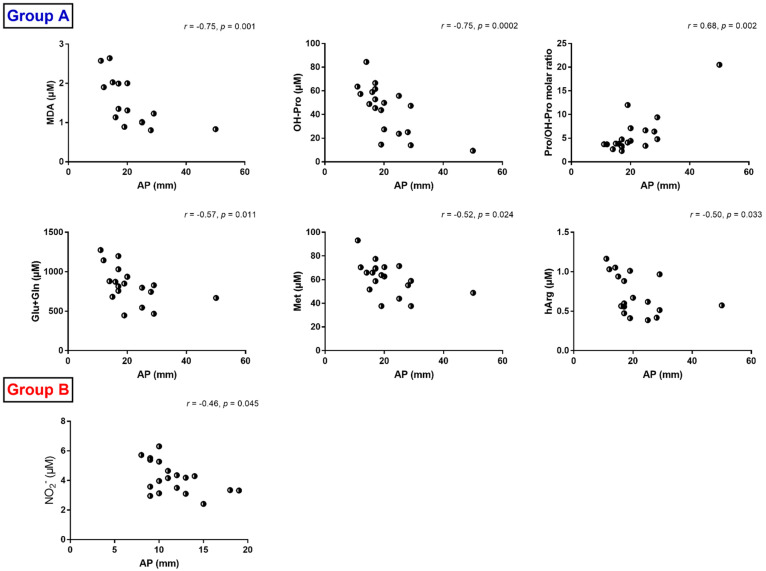
Illustration of statistically significant correlations (*r* > 0.4, *p* < 0.05) found between AP diameter and serum concentration of the indicated metabolites in Group A (surgical treatment) and Group B (conservative treatment). Spearman correlation was performed in all comparisons.

We tested for potential correlations between clinical parameters and serum biomarkers in the UPJO patients. Of particular interest was the preoperative diameter of the renal pelvis anterior–posterior (AP), which was higher (by 36%) in Group A compared to Group B (*p* = 0.02) (Figure 5). In Group B, there was a moderate inverse correlation between AP diameter and serum nitrite concentration (*r* = −0.46, *p* = 0.045). In contrast, in Group A, the AP diameter strongly correlated with MDA (*r* = −0.75, *p* = 0.001), OH-Pro (*r* = −0.75, *p* = 0.0002) and the Pro/OH-Pro molar ratio (*r* = 0.68, *p* = 0.002) and moderately with Glu + Gln (*r* = −0.57, *p* = 0.011), Met (*r* = −0.52, *p* = 0.024) and hArg (*r* = −0.50, *p* = 0.033) (Figure 5). MDA and thromboxane A_2_ were concomitantly produced from unsaturated fatty acids including arachidonic acid, in part by the action of cyclooxygenase (COX) [22]. OH-Pro was produced enzymatically by the ascorbic acid-dependent hydroxylation of proline residues in proteins, notably in collagen [33]. The strong inverse correlations between the preoperative AP diameter and the serum concentrations of MDA and OH-Pro might suggest that the UPJO patients of Group A had diminished lipid peroxidation and proline hydroxylation rates.

## 4. Discussion

### 4.1. General Issues

Since hydronephrosis is a common finding in prenatal ultrasound, and even if imaging techniques successfully detect asymptomatic cases, the emergent question of progression to severe renal impairment remains crucial for pediatric patient stratification and management [7].

Obstructive hydronephrosis, predominantly arising from UPJO, may result in chronic kidney disease (CKD) in children [25]. Longstanding complications of renal damage may appear later, in young adulthood, and by that time, noxious effects of renal development could have happened [7]. The modern era of minimally invasive approaches intensifies the need to discover reliable and sensitive diagnostic and prognostic biomarkers for the most effective and refined treatment of UPJO patients.

Longitudinal measures of common clinical parameters may serve in the prediction of progression of CKD from obstructive uropathy (OU) at an age-specific baseline [34]. Metabolomics and proteomics studies have also contributed to both serum and urinary [7,8,9,10,35,36,37] biomarkers’ pool, but they still have not been established in clinical practice.

The kidney profoundly affects the metabolome with respect to glomerular filtration, tubular secretion and reuptake and catabolism of amino acids [38]. Reliable quantitative measurement of amino acids and their metabolites in biological samples such as blood and urine in health and disease is a useful analytical tool to establish reference values and ranges and to monitor metabolic perturbations, nutritional interventions, treatment efficacy and renal drug toxicity in disease management [39]. Thus far, two metabolomics-based studies have been conducted on pediatric UPJO by analyzing the urinary [9] or circulating [10] metabolome. Our previous study, using HILIC–MS/MS of pediatric UPJO patients [10], revealed alterations in some serum amino acids and their metabolites. In serum samples of the same UPJO patients, we measured the concentrations of amino acids and selected metabolites, notably of those involved in L-arginine pathways, by using orthogonal GC– MS methods for amino acids [29], nitrite and nitrate, the metabolites of L-arginine-derived NO and MDA, presently the most useful biomarker of oxidative stress [22]. The aim of the study was to investigate whether the metabolic amino acid profile could distinguish between the two groups of UPJO patients, i.e., 21 surgical and 21 conservative UPJO patients, as well as between the group of 19 age-matched healthy children. Such distinction is of particular importance, notably in early disease stages, possibly avoiding time-consuming, invasive and expensive laboratory tests.

### 4.2. Homoarginine, Asymmetric Dimethylarginine, Guanidinoacetate and Malondialdehyde

The serum concentrations of hArg and ADMA were found to be statistically significantly higher in the UPJO patients of Group B (conservatively treated patients) compared to the UPJO patients of Group A (surgically treated). The serum hArg concentration in Group B was also higher than in Group C, i.e., in the healthy infants/children. In adults, low circulating and low excretion rates of hArg are considered risk factors in various diseases including CKD [29,40,41,42,43]. The plasma mean concentration of hArg was measured to be 0.54 µM in 106 pre-term newborns, with the concentration being higher in the boys (0.63 µM) compared to the girls (0.47 µM) [44]. Higher mean plasma hArg concentrations were found in 54 healthy children with a mean age of 11 years [45], suggesting a considerable increase in the first 11 years of life. The serum concentrations of ADMA in the healthy infants of the present study are comparable to those measured in plasma of pre-term neonates [44] and in healthy infants in the first 2 years of life [46], but higher than the plasma concentrations in 54 healthy children of a mean age of 11 years 0.6 µM [45], suggesting a considerable increase in the first 11 years of life.

Obviously, the biosynthesis rates of hArg and ADMA change in an opposite manner during the first years of life of humans. In pre-term neonates, the mean hArg-ADMA-molar ratio (hArg/ADMA) was about 0.65 [44], whereas the hArg/ADMA molar ratio in healthy children was 3.4 [45]. In the present study, the serum hArg/ADMA molar ratio was about 1, which was closer to the pre-term neonates [44].

In adults, lower circulating concentrations of hArg and higher circulating ADMA concentrations are considered risk factors in the renal and cardiovascular systems [29,40]. Yet, the importance of hArg and ADMA in infants and children is uncertain and remains to be investigated. Homoarginine, a non-proteinogenic amino acid, is primarily synthesized in the kidneys from Arg and Lys via L-arginine:glycine amidinotransferase (AGAT) activity, wherein the last is also involved in creatine metabolism, catalyzing the conversion of Arg and Gly into guanidino acetate (GAA) and Orn [29,40].

In the present study, the molar ratios of the serum concentrations of GAA and hArg in all three groups were very similar and of the order of 1:1, whereas the mean GAA–to guanidino acetate hArg molar ratio in kidney-transplanted and immunosuppressed children (mean age, about 14 years) was 1.3:1 [29]. This may suggest relatively constant AGAT-catalyzed synthesis rates of hArg and GAA from birth to the age of 14 years.

The methylation of protein-bound arginine forms the endogenous arginine analogue ADMA [47]. Both ADMA and its stereoisomer, symmetric dimethylarginine (SDMA), are excreted in urine, while only ADMA is subjected to hydrolytic degradation to citrulline and dimethylamine in the kidney or in the liver [48,49]. The altered dimethylamine that was found to be different between the studied groups in our previous study [10] could also be linked to choline as a breakdown product via its formation to trimethylamine [45,47,50].

The serum concentration of MDA was found to be statistically significantly higher in the UPJO patients of Group B (conservatively treated patients) compared to the UPJO patients of Group A (surgically treated patients), with the serum MDA concentration in Group A being lower than in Group C. MDA results from lipid peroxidation of polyunsaturated fatty acids [22,51,52,53]. Oxidative stress is immediately related to the pathophysiology of several kidney diseases, while many of the complications of these diseases are caused by oxidative stress, intermediate metabolites or inflammation [54].

It is interesting to note that the differences of the serum concentrations between Group A and Group B were highest for MDA (56.7%), ADMA (34%) and hArg (36%) followed by GAA (14.8%), with almost no appreciable differences between the groups regarding Gly (8.6%), Asn + Asp (6%), Arg (4.5%), nitrate (10.5%) or nitrite (7.2%). Based on our findings, the conservative group demonstrated profound elevated indices of oxidative stress despite the clinical severity of Group A.

### 4.3. Nitrite and Nitrate

In human body, L-arginine is oxidized to NO by nitric oxide synthase (NOS). Nitrite and nitrate are major metabolites of NO. Under standardized conditions, notably including abstinence from a nitrate/nitrite-rich diet, circulating and excretory nitrite and nitrate may serve as surrogates for local, systemic and whole-body NO synthesis in health and disease and in clinical studies [32,55]. The serum concentrations of nitrite and nitrate in the UPJO patients and in the healthy controls of the present study are comparable in these groups and comparable to those reported in other pediatric studies, suggesting unaltered systemic NO synthesis in the UPJO patients. The serum arginine concentrations were even higher in the UPJO patients, suggesting sufficient availability of L-arginine, the substrate of all known NOS isozymes. Yet, this does not exclude an altered L-arginine/NO pathway in the UPJO patients.

### 4.4. Correlation between Metabolomics and Clinical Data

In order to investigate the metabolic effect of obstructive hydronephrosis, Spearman correlations were performed as a “nonparametric measure of rank correlation” between metabolomics and the clinical parameters of UPJO, such as increased BUN, SCr and increased AP diameter. Multiple (and in cases, strong) correlations were found among serum concentrations of amino acids/derivatives and the aforementioned biochemical parameters as well as patients’ characteristics. Among the conventional circulating biomarkers of renal function such as BUN and serum creatinine [56], the first one presented twelve correlations in the surgically treated group. BUN refers to the serum non-protein nitrogenous waste product of protein metabolism. Ammonia is produced by the deamination of amino acids derived from protein catabolism followed by its conversation to nontoxic urea in the liver and excretion in urine [57,58,59]. Interestingly, glutamate and methionine, as measured using the previously applied HILIC–MS/MS method [10], were also strongly correlated with BUN in the surgically treated group among other significant correlations (data not shown). More specifically, BUN demonstrated a strong negative correlation with glutamate (*r* = −0.62, *p* = 0.005) and a moderate negative correlation with methionine (*r* = −0.46, *p* = 0.047). Furthermore, BUN in the conservative group was correlated with gut microbiome-derived choline metabolism, i.e., choline, DMA, trimethylamine *N*-oxide (TMAO) and homocysteine. The effect of choline on health is very important, with significant meaning at the various stages of development, since there is even a different maternal and offspring response to the different forms of consumed choline [60].

On the other hand, AP diameter, which is considered to be one of the crucial parameters indicating surgical correction [61], as an increased AP diameter means decreased renal parenchyma and so possibly affected renal function, was correlated to several amino acids and derivatives, namely OH-Pro, Glu + Gln, Met, hArg and Pro/OH-Pro molar ratio. In our previous study, AP was also negatively correlated with glutamate (*r* = −0.59, *p* = 0.008), methionine (*r* = −0.52, *p* = 0.022) and creatinine (*r* = −0.56, *p* = 0.013).

### 4.5. Comparison between GC-MS and HILIC-MS/MS

GC–MS and HILIC–MS/MS analysis combined can widen the analytical coverage of biochemical pathways. Nitrite and nitrate are best analyzed by GC–MS [62]. On the other hand, the GC–MS method used in the present study does not discriminate between asparagine and aspartate, between citrulline and ornithine, between glutamine and glutamate (because of interconversion) nor between leucine and isoleucine (because of co-elution). Compared to our previously reported results obtained by HILIC–MS/MS [24], no significant differences were found for Asp + Asn or Ile + Leu in the present study using GC–MS. Furthermore, HILIC–MS/MS results showed Glu to be significantly altered between Group B and Group C; in GC–MS the sum of Gln and Glu was not proven to be significantly altered, presenting only a difference of 26.7% between Group A and Group B. Glu and ammonia from renal Gln are complementary to hepatic urea synthesis and are involved in several biochemical pathways [48].

Gly and Phe were also found to differ between the groups when measured by HILIC–MS/MS but not when measured by GC–MS. In the present study, serum Tyr concentrations differed by 22.7% between Group A and Group B (lower in Group A), but the difference was not statistically significant. In chronic renal failure in elderly adults, plasma Tyr concentrations were lower than in healthy adults [38].

### 4.6. Study Limitations

Potential limitations of our study are the relatively small number of conservatively and surgically managed UPJO infants as well as of healthy controls. Another potential limitation is that only circulating biochemical parameters were analyzed. Serum concentrations of amino acids, their metabolites and of the oxidative stress biomarker MDA provide important information.

## 5. Conclusions and Perspectives

The results of the present study observed by GC–MS and those of a previous study observed by HILIC–MS/MS, i.e., by using two orthogonal analytical techniques on serum samples, revealed in part confirmative and in part complementary observations and potential biomarkers that are considered to help better understand the underlying biochemical mechanisms and to lead to an improvement of the clinical situation of UPJO infants by surgical and potentially by therapeutic treatment. Groups A and B were found to differ preoperatively both in AP diameter (by 36%) and in its correlations with certain serum metabolites in both techniques.

By using GC–MS-based metabolomics and on the basis of differences in serum concentrations of amino acids and their metabolites, as well as in differences in their correlations, remarkable systemic alterations of L-arginine-involving pathways and of oxidative stress were observed in the two groups of infants with UPJO. The L-arginine metabolites hArg (AGAT pathway), ADMA (PRMT/DDAH/NOS pathways) and MDA (polyunsaturated fatty acids peroxidation) are likely to be linked to vital renal processes. Serum hArg, ADMA and MDA seem to be useful biomarkers in UPJO. Measurement of these biomarkers in urine, a non-invasive procedure, is warranted. Such measurements may provide more renal-relevant information and, in combination with other circulating biomarkers, may contribute to improve the management of UPJO. Circulating hArg and ADMA are assumed to have opposite importance in adults, with high hArg concentrations and low ADMA concentrations being favorable to health [63]. It is worthy of mention that hArg and ADMA seem to play major roles in pregnancy, with alterations potentially leading to severe disease such as preeclampsia and disadvantageous birth outcome [44,64,65,66,67]. A study extending our results could further investigate the potential beneficial effects of supplementation of amino acids including L-arginine (commonly deficient) and hArg as well as antioxidants and cofactors such as ascorbic acid (vitamin C) during pregnancy on UPJO-induced renal dysfunction. Such investigations are warranted and should preferably include measurement of circulating and urinary amino acids and selected metabolites by the use of GC–MS- and HILIC–MS/MS-based approaches.

## Figures and Tables

**Table 1:** Demographic and clinical characteristics of the UPJO patients and controls of the present study.

Characteristics	Group A (*n* = 21)	Group B (*n* = 21)	Group C (*n* = 19)	*p* Value
Gender (male/female)	19/2	16/5	11/8	N.A.
Laterality (right/left)	10/11	1/20	N.A.	N.A.
Diagnosis (pre-/postnatally)	20/1	21/0	N.A.	N.A.
Age at admission in the study (months) (median (interquartile range))	2 (1–5.3)	2 (2–6)	2 (1–2.5)	0.89
Preoperative anterior–posterior (AP) renal pelvic diameter (mm) (median (interquartile range))	18 (16.8–25)	11.5 (9.8–13)	N.A.	0.02
Preoperative anterior–posterior (AP) renal pelvic diameter (SFU grade)	grade 3:9 grade 4:12	grade 1:4 grade 2:17	N.A.	N.A.
Serum creatinine (SCr) (mg/dL) (median (interquartile range))	0.49 (0.45–0.53)	0.48 (0.43–0.50)	0.52 (0.44–0.55)	0.09
Blood urea nitrogen (BUN) (mg/dL) (median (interquartile range))	20 (15.5–24.5)	15 (11.5–17.5)	13 (11–25)	0.32
MAG-3				
T1/2 > 20 min T1/2: 15—20 min T1/2 < 15 min	16 5 0	0 0 21	N.A.	N.A.

Abbreviations: UPJO, ureteropelvic junction obstruction; N.A., not applicable; SFU, Society for Fetal Urology; MAG, mercaptoacetyltriglycine; T1/2, half-life.

**Table 2:** Serum concentrations (µM) ^a^ of the analytes and serum molar ratios of Pro/OH-Pro and hArg/ADMA in the examined groups.

Analyte	Group A (*n* = 21)	Group B (*n* = 21)	Group C (*n* = 19)	A vs. B (*q* Value)	A vs. C (*q* Value)	B vs. C (*q* Value)
Alanine (Ala)	407 (378–481)	515 (416–654)	416 (345–534)	0.102	0.999	0.102
Threonine (Thr)	217 (167–239)	241 (199–297)	286 (178–395)	0.275	0.129	0.519
Glycine (Gly)	301 ± 62.8	316 ± 107	325 ± 167	0.999	0.999	0.999
Valine (Val)	263 ± 51.4	300 ± 95.2	293 ± 144	0.602	0.602	0.885
Serine (Ser)	212 (200–234)	230 (183–308)	231 (192–786)	0.444	0.444	0.768
Sarcosine	1.09 (0.92–1.76)	1.47 (0.97–1.94)	1.32 (1.12–2.0)	0.373	0.373	0.999
Leucine + Isoleucine (Leu + Ile)	250 (226–294)	278 (224–319)	246 (226–349)	0.752	0.752	0.752
Guanidino acetate (GAA)	1.09 (1.02–1.15)	1.33 (0.85–1.67)	1.01 (0.81–1.23)	0.397	0.397	0.236
Aspartate + Asparagine (Asp + Asn)	74.5 (66.5–91.8)	76.2 (59.4–102)	77.6 (65.9–112)	0.903	0.903	0.903
4-hydroxy-proline (OH-Pro)	48.9 (26.3–58.2)	41.7 (32.1–67.7)	44.2 (20.0–71.8)	0.901	0.901	0.901
Proline (Pro)	192 (171–218)	227 (174–273)	234 (169–267)	0.165	0.165	0.935
Methionine (Met)	61.8 ± 13.5	71.9 ± 15.0	67.0 ± 19.1	0.186	0.367	0.367
Glutamate + Glutamine (Glu + Gln)	829 (714–937)	963 (790–1236)	939 (728–1264)	0.261	0.437	0.552
Ornithine + Citrouline (Orn + Cit)	109 (91.2–148)	116 (85.1–212)	132 (71.1–185)	0.839	0.839	0.890
Phenylalanine (Phe)	99.1 ± 20.8	111 ± 43.1	106 ± 56.5	0.763	0.763	0.763
Tyrosine (Tyr)	101 ± 25.4	125 ± 49.7	109 ± 56.7	0.377	0.629	0.474
Lysine (Lys)	209 (169–240)	224 (172–281)	198 (159–302)	0.851	0.851	0.851
Arginine (Arg)	130 ± 40.8	141 ± 49.5	112 ± 64.1	0.560	0.486	0.318
homoarginine (hArg)	0.67 ± 0.22	1.05 ± 0.43	0.76 ± 0.29	**0.006**	0.261	**0.014**
Asymmetric dimethylarginine (ADMA)	0.85 ± 0.17	1.29 ± 0.71	1.20 ± 0.64	**0.050**	0.140	0.650
Nitrate	69.1 (64.7–80.3)	75.6 (62.8–87.0)	71.9 (67.1–95.9)	0.437	0.437	0.941
Nitrite	4.3 (3.0–5.9)	4.2 (3.3–4.8)	4.3 (3.4–5.7)	0.865	0.865	0.865
Malondialdehyde (MDA)	1.5 ± 0.6	3.4 ± 3.2	2.4 ± 1.4	**0.022**	0.188	0.188
Pro/OH-Pro	4.1 (3.5–6.5)	4.0 (3.2–7.6)	4.9 (2.3–6.3)	0.999	0.999	0.999
hArg/ADMA	0.74 ± 0.22	1.1 ± 0.65	0.74 ± 0.37	0.064	0.909	0.065

^a^ Mean ± standard deviation or median (interquartile range) in the case of normally or non-normally distributed data, respectively. Significant comparisons are marked in bold.

**Table 3:** Statistically significant metabolites highlighted after comparison of the examined groups. area under the curve (AUC) and their respective 95% confidence interval (CI) values and logarithm base two of the fold change (FC).

	A vs. B		B vs. C	
	AUC (95% CI)	log_2_FC	AUC (95% CI)	log_2_FC
ADMA	0.66 (0.48–0.84)	0.6	–	
hArg	0.72 (0.56–0.88)	0.65	0.68 (0.51–0.87)	−0.47
MDA	0.64 (0.47–0.82)	1.21	–	

## Data Availability

The data presented in this study are available on request from the corresponding author. The data are not publicly available due to privacy.

## Supplementary Materials

The following are available online at https://www.mdpi.com/article/10.3390/jcm10071467/s1, additional information regarding the GC–MS analysis of amino acids, metabolites, nitrite, nitrate and MDA, Table S1: Summary of the analytes and the respective internal standards, their *m*/*z* values used in SIM and the retention times (RT) of the derivatives observed in GC–MS analyses, Figure S1: Total ion chromatograms from GC-MS analyses (A) of amino acids and selected metabolites, and (B) of nitrate, nitrite, creatinine and malondialdehyde in a pooled serum sample.
