# Supplementary material for: Diminished Systemic Amino Acids Metabolome and Lipid Peroxidation in Ureteropelvic Junction Obstruction (UPJO) Infants Requiring Surgery
Source: J Clin Med. 2021 Apr 2;10(7):1467. doi: 10.3390/jcm10071467 (PMC8038180; doi:10.3390/jcm10071467)
Supplement: Supplementary file 1 [file jcm-10-01467-s001.pdf]

Supplementary Materials

Diminished Systemic Amino Acids Metabolome and Lipid Peroxidation in Ureteropelvic Junction Obstruction (UPJO) Infants Requiring Surgery.

Olga Begou[1,2,3‡*], Antigoni Pavlaki[4‡], Olga Deda[2,5], Alexander Bollenbach[3], Kathrin Drabert[3], Helen Gika[2,5], Evangelia Farmaki[6], John Dotis[4], Nikoleta Printza[4], Georgios Theodoridis[1,2], Dimitrios Tsikas[3]

[1] Laboratory of Analytical Chemistry, Department of Chemistry, Aristotle University of Thessaloniki, University Campus, 54124, Thessaloniki, Greece; mpegolga@chem.auth.gr (O.B.); gtheodor@chem.auth.gr (G.T.)

[2] Biomic_AUTh, Center for Interdisciplinary Research and Innovation (CIRI-AUTH), Balkan Center, B1.4, 10th km Thessaloniki-Thermi Rd, P.O. Box 8318, 57001, Thessaloniki, Greece; mpegolga@chem.auth.gr (O.B.); oliadmy@gmail.com (O.D.); gkikae@auth.gr (H.G.); gtheodor@chem.auth.gr (G.T.)

[3] Core Unit Proteomics, Hannover Medical School, Institute of Toxicology, Carl-Neuberg-Strasse 1, 30625, Hannover, Germany; mpegolga@chem.auth.gr (O.B.); bollenbach.alex@gmail.com (A.B.); drabert.kathrin@mh-hannover.de (K.D.); tsikas.dimitros@mh-hannover.de (D.T.)

[4] Paediatric Nephrology Unit, First Department of Paediatrics, Hippokratio Hospital, Aristotle University of Thessaloniki, University Campus, 54124, Thessaloniki, Greece; antigonipavlaki@yahoo.gr (A.P.); yan_dot@yahoo.com (J.D.); nprintza@auth.gr (N.P.)

[5] Laboratory of Forensic Medicine and Toxicology, School of Medicine, Aristotle University of Thessaloniki, University Campus, 54124, Thessaloniki, Greece; oliadmy@gmail.com (O.D.); gkikae@auth.gr (H.G.)

[6] Paediatric Immunology and Rheumatology Referral Center, First Department of Paediatrics, Hippokratio Hospital, University Campus, 54124, Thessaloniki, Greece; farmakg@auth.gr (E.F.)

* Correspondence: mpegolga@chem.auth.gr ; +30-2310 990596

‡ These authors contributed equally and are both first authors

Sample Preparation for Amino Acids and their Metabolites

Frozen (-20 °C) serum samples were allowed to thaw at room temperature and then were vortex-mixed for 5 min. Sample preparation was based on a previously reported two-step procedure [21]. In brief, 10-µL aliquots of serum were treated with 50-µL aliquots of a 2 M HCl/methanol (MeOH) solution for protein precipitation. The sample were vortex-mixed for 2 min and then centrifuged at 4,500 ×g, and 4 °C for 5 min. Forty microliters of the clear supernatant were evaporated to dryness under a gentle stream of nitrogen gas and the dry residue was reconstituted in 100 µL of 2 M HCl in MeOH. The resulting sample was vigorously vortex-mixed for 2 min and finally placed in a thermoblock at 80 °C for 1 h (step). Meanwhile, 20-µL aliquots of the internal standard mixture of all analytes analysed were evaporated to dryness, the residues were reconstituted in 500-µL aliquots of 2 M HCl/CD_3OD, the samples were vortex-mixed and placed in a thermoblock at 80 °C for 1 h (step 2).

After cooling down to room temperature, 10-µL aliquots of the samples derived from step 2 were added to the derivatized samples from step 1. The final mixtures were then evaporated to dryness under a stream of nitrogen gas and the dry residues were reconstituted in 100-µL aliquots of freshly prepared pentafluoropropionic anhydride-ethyl acetate (PFPA-EA, 1:4, v/v) solution. After vortexing for 2 min, the samples were placed in a thermoblock at 65 °C for 30 min. Then the final derivatization step, the resulting mixtures were evaporated to dryness and the residues were reconstituted in 400-µL aliquots of borate buffer-toluene (1:1, v/v). This step was followed by vortexing for 1 min and centrifugation at 4,500 ×g and 4 °C for 5 min. Aliquots (150 µL) of the final extracts were transferred to autosampler GC-MS glass vials which were sealed and subjected to GC-MS analysis.

Sample Preparation for Nitrite, Nitrate and MDA

Frozen (-20 °C) serum samples were left to thaw at room temperature and were vortex-mixed for 1 min. Sample preparation was adopted from Hanff *et al.* [22]. Briefly, to 100-µL aliquots of serum were added 10-µL aliquots of a mixture of $^{15}NO_2^-$ and $^{15}NO_3^-$, 15.4-µL aliquots of d_2-MDA (65 µM), 400-µL aliquots of acetone and 10-µL aliquots of PFB-Br. The resulting samples were vortex-mixed for 2 min and then placed in a thermoblock at 50 °C for 1 h. After cooling down to room temperature, solvents were evaporated under a stream of nitrogen for 5 min (4 psi) and thereafter, 1000-µL aliquots of EA were added. The samples were vortex-mixed and centrifuged at 4,500 ×g for 5 min. Small amounts of solid anhydrous Na_2SO_4 were added to 900-µL aliquots of the clear supernatant. After centrifugation, 750-µL aliquots of the final extracts were transferred to autosampler GC-MS glass vials which were sealed and placed in the autosampler and subjected to GC-MS analysis.

GC-MS Conditions

Quantification of all analytes was performed on a single quadrupole mass spectrometer model ISQ (ThermoFisher, Dreieich, Germany) in the selected-ion monitoring (SIM) mode. Helium, at a constant flow of 1 mL/min, was selected as the carrier gas, whereas methane at a flow rate of 2.4 mL/min was used as the reagent gas for NICI. Electron energy was set to 70 eV and electron current to 50 µA.

The following oven temperature program was used for amino acids: 0.5 min at 40 °C, then increased to 210 °C at a rate of 15 °C/min and then to 320 °C at a rate of 35 °C/min for 1 min. Injector and ion-source were kept constant at 280 °C and 250 °C, respectively. The dwell-time was set at 100 ms for each ion. The m/z values used in SIM of the amino acids are given in **Table S1**.

The following oven temperature program was used for nitrite, nitrate and MDA: 1 min at 70 °C, then increased to 250 °C at a rate of 30 °C/min and then to 320 °C at a rate of 70 °C/min for 1 min. The dwell-time was set at 50 ms for each ion. The m/z values used in SIM of the nitrite, nitrate and MDA are given in **Table S1**.

The concentration of the analytes in study serum samples was calculated by the equation:

$$C_x = C_{IS} \times PAR$$

whereas: C_x is the concentration of the measured analyte in the serum sample, C_{IS} is the known concentration of the respective internal standard (IS) added to the serum sample, and PAR is the peak area ratio, i.e., the ratio of the peak area of the analyte to the peak area of the respective internal standard.

Table S1. Summary of the analytes and the respective internal standards, their *m/z* values used in SIM, and the retention times (RT) of the derivatives observed in GC-MS analyses

Analyte	*m/z*	RT (min)	Analyte	*m/z*	RT (min)
**Alanine**	229	3.52	**Glutamine/Glutamate**	301	7.68
**d_3-Alanine**	232	3.50	**d_3-Glutamine/Glutamate**	307	7.64
**Threonine**	259	3.90	**Ornithine/Citrulline**	418	8.30
**d_3-Threonine**	262	3.87	**d_3-Ornithine/Citrulline**	421	8.30
**Glycine**	215	4.0	**Phenylalanine**	305	8.47
**d_3-Glycine**	218	4.0	**d_3-Phenylalanine**	308	8.45
**Valine**	257	4.21	**Tyrosine**	233	8.80
**d_3-Valine**	260	4.19	**d_3-Tyrosine**	236	8.78
**Serine**	207	4.25	**Lysine**	432	9.25
**d_3-Serine**	210	4.23	**d_3-Lysine**	435	9.23
**Sarcosine**	229	4.73	**Arginine**	586	9.37
**d_3-Sarcosine**	232	4.71	**d_3-Arginine**	589	9.35
**Leucine/Isoleucine**	271	4.86	**Homoarginine**	600	10.15
**d_3-Leucine/Isoleucine**	274	4.84	**d_3-Homoarginine**	603	10.13
**Guanidino acetic acid**	383	6.46	**Tryptophan**	233	11.18
**d_3-Guanidino acetic acid**	386	6.44	**d_3-Tryptophan**	236	11.15
**Asparagine/Aspartate**	287	6.49	**Asymmetric dimethylarginine**	634	11.54
**d_3-Asparagine/Aspartate**	293	6.45	**d_3-Asymmetric dimethylarginine**	637	11.50
**4-Hydroxy-proline**	397	6.62			
**d_3-4-Hydroxy-proline**	400	6.59	**Nitrate**	62	3.12
**Proline**	255	6.93	**^{15}N-Nitrate**	63	3.12
**d_3-Proline**	258	6.91	**Nitrite**	46	3.22
**Methionine**	289	7.62	**^{15}N-Nitrite**	47	3.22
**d_3-Methionine**	292	7.60	**Malondialdehyde**	251	6.79
			**d_2-Malondialdehyde**	253	6.79

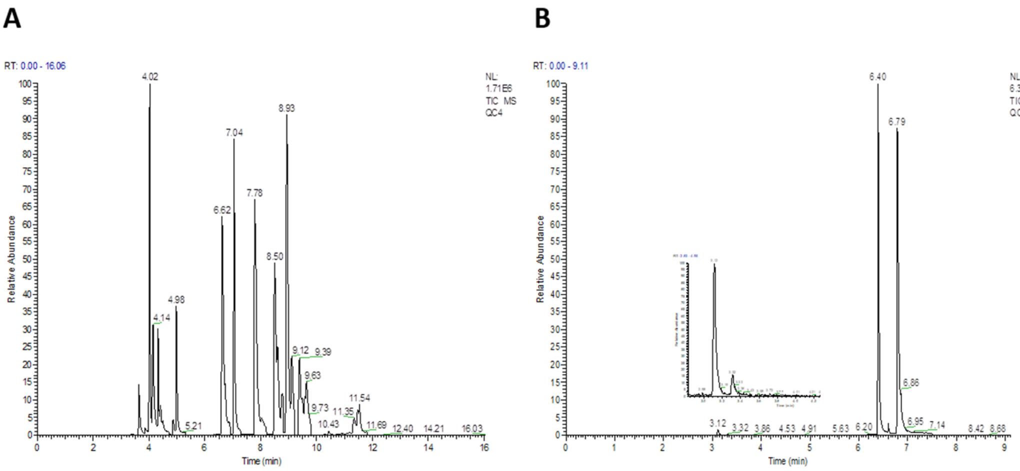

Figure S1. Total ion chromatograms from GC-MS analyses (A) of amino acids and selected metabolites, and (B) of nitrate, nitrite, creatinine and malondialdehyde in a pooled serum sample. The inset in (B) shows the derivatives of nitrate and nitrite. The numbers above the peaks indicate the retention times of the analytes. The *m/z* values used in SIM of the analytes derivatives and their retention times are summarized in Table S1.

Quality Control

Concomitantly analyzed quality control (QC) serum samples (n=12) were used for the determination of the precision of the GC-MS methods for amino acids and metabolites, as well as for nitrate, nitrite and MDA. The precision was expressed in terms of relative standard deviation (RSD, %). The precision of the GC-MS method for amino acids ranged between 0.9 % (threonine) and 19.9 % (sarcosine). The precision of the GC-MS method was 2.2 % for nitrate, 11.4 % for nitrite, and 14.5 % for MDA. These data underlie the precision of both GC-MS methods.

References

21. Hanff, E.; Ruben, S.; Kreuzer, M.; Bollenbach, A.; Kayacelebi, A.A.; Das, A.M.; Versen-Höynck, F. von; Kaisenberg, C.S. von; Haffner, D.; Ückert, S.; et al. Development and Validation of GC–MS Methods for the Comprehensive Analysis of Amino Acids in Plasma and Urine and Applications to the HELLP Syndrome and Pediatric Kidney Transplantation: Evidence of Altered Methylation, Transamidination, and Arginase Activity. *Amino Acids* **2019**, *51*, 529–547, doi:10.1007/s00726-018-02688-w.

22. Hanff, E.; Eisenga, M.F.; Beckmann, B.; Bakker, S.J.L.; Tsikas, D. Simultaneous Pentafluorobenzyl Derivatization and GC-ECNICI-MS Measurement of Nitrite and Malondialdehyde in Human Urine: Close Positive Correlation between These Disparate Oxidative Stress Biomarkers. *J Chromatogr B Analyt Technol Biomed Life Sci* **2017**, *1043*, 167–175, doi:10.1016/j.jchromb.2016.07.027.

23. Gika, H.G.; Zisi, C.; Theodoridis, G.; Wilson, I.D. Protocol for Quality Control in Metabolic Profiling of Biological Fluids by U(H)PLC-MS. J. Chromatogr B Analyt Technol Biomed Life Sci 2016, 1008, 15–25, doi:10.1016/j.jchromb.2015.10.045.

24. Begou, O.; Gika, H.G.; Theodoridis, G.A.; Wilson, I.D. Quality Control and Validation Issues in LC-MS Metabolomics. Methods Mol Biol 2018, 1738, 15–26, doi:10.1007/978-1-4939-7643-0_2.
